# Psilocin, LSD, mescaline, and DOB all induce broadband desynchronization of EEG and disconnection in rats with robust translational validity

**DOI:** 10.1038/s41398-021-01603-4

**Published:** 2021-10-02

**Authors:** Čestmír Vejmola, Filip Tylš, Václava Piorecká, Vlastimil Koudelka, Lukáš Kadeřábek, Tomáš Novák, Tomáš Páleníček

**Affiliations:** 1 National Institute of Mental Health, Klecany, Czechia; 2 Third Faculty of Medicine, Charles University, Prague, Czechia; 3 Department of Biomedical Technology, Faculty of Biomedical Engineering, Czech Technical University in Prague, Prague, Czechia

**Keywords:** Neuroscience, Pharmacodynamics, Psychiatric disorders

## Abstract

Serotonergic psychedelics are recently gaining a lot of attention as a potential treatment of several neuropsychiatric disorders. Broadband desynchronization of EEG activity and disconnection in humans have been repeatedly shown; however, translational data from animals are completely lacking. Therefore, the main aim of our study was to assess the effects of tryptamine and phenethylamine psychedelics (psilocin 4 mg/kg, LSD 0.2 mg/kg, mescaline 100 mg/kg, and DOB 5 mg/kg) on EEG in freely moving rats. A system consisting of 14 cortical EEG electrodes, co-registration of behavioral activity of animals with subsequent analysis only in segments corresponding to behavioral inactivity (resting-state-like EEG) was used in order to reach a high level of translational validity. Analyses of the mean power, topographic brain-mapping, and functional connectivity revealed that all of the psychedelics irrespective of the structural family induced overall and time-dependent global decrease/desynchronization of EEG activity and disconnection within 1–40 Hz. Major changes in activity were localized on the large areas of the frontal and sensorimotor cortex showing some subtle spatial patterns characterizing each substance. A rebound of occipital theta (4–8 Hz) activity was detected at later stages after treatment with mescaline and LSD. Connectivity analyses showed an overall decrease in global connectivity for both the components of cross-spectral and phase-lagged coherence. Since our results show almost identical effects to those known from human EEG/MEG studies, we conclude that our method has robust translational validity.

## Introduction

Serotonergic psychedelics are drugs that dramatically affect human perception, cognition and emotions, and induce so-called “altered state of consciousness” [1]. According to their binding core structure, psychedelics may be divided into two main chemical classes—tryptamine derivatives (e.g., lysergic acid diethylamide (LSD), psilocybin, N,N-dimethyltryptamine (DMT), 5-methoxy-dimethyltryptamine (5-MeO-DMT)) [2, 3], and phenethylamine derivatives (e.g., mescaline, 2,5-dimethoxy-4-bromoamphetamine (DOB)) [4]. Despite the structural difference, they produce very similar effects, which are mediated mainly via their agonistic action on 5-HT2A receptors [5, 6]. Over the last decade, psychedelics have gained popularity mainly for their rapid onset and long-lasting effects in the treatment of depression [7–9]. One of the hypothesized mechanisms underlying their unique and rapid effects is explained by dramatically switching the brain functional connectivity states (for more see refs. [10–12]) which may be evaluated by functional neuroimaging methods such as PET, fMRI, or EEG.

In animal models, the effects of psychedelics cannot be examined in the same way as in humans, as animals obviously lack the ability to verbally share their experiences. Therefore, in order to describe the phenomenology of the experience in animal studies scientists have to deal with behavioral experiments, such as evaluation of locomotion, exploratory behavior, sensorimotor processing, stereotyped behaviors, cognitive tasks, etc., which have all shown to be altered after psychedelics, but often have a limited translational validity to humans [13–19]. Until now we still do not know whether psychedelics induce visual perceptual changes in animals such as illusions and hallucinations, effects that are fundamental in humans [20]. Evaluating the electrical activity of the brain, in contrast to behavioral models, examines the biological processes of the same origin and very likely the same function. Although a fairly clear picture of QEEG finding is known in humans, it is not yet available in animals. EEG, unlike other methods, can be used as a whole-brain neuroimaging tool in awake and freely moving animals.

Human EEG studies with serotonergic psychedelics consistently report a broadband spectral power decrease (delta to gamma) most pronounced within the alpha band (8–12 Hz) and a decrease in functional connectivity and integrity of networks [21–26]. On the other hand, increases in higher frequencies (gamma oscillations, 30 Hz and above) have been also described [27–29]; however, the effects are hard to interpret due to typical contamination related to increased tension of the facial muscles. MEG, in contrast to EEG, is devoid of this contamination [30], and on the contrary shows a decrease in oscillations within the gamma range [31].

Early animal EEG studies with psychedelics used incomparable conditions to human research. While human EEG was typically recorded during resting state with eyes opened or closed, studies in animals were performed in anesthetized or immobilized subjects. Despite these limitations, an overall effect in reducing the amplitude and desynchronizing the signal was mostly observed [32–36]. Recent psychedelic studies in animal models are scarce and mainly focused on specific areas of the brain, e.g., recording local field potentials (LFPs). Nevertheless, they also showed the general trend of psychedelics to decrease the power of low- [37, 38] and high-frequency oscillations [39, 40].

The main aim of our study was to describe the global EEG changes induced by psilocin, LSD, mescaline, and DOB in freely moving rats. In order to make the study as translational as possible, we recorded the signal from 14 cortical surface electrodes implanted on the frontal, parietal, and temporal areas and we further analyzed only the segments of EEG signal corresponding to behavioral inactivity, as a model of resting-state-like EEG [41]. The obtained data were then subjected to 3D spline mapping of EEG and computation of connectivity using cross-spectral and phase-lagged coherence. Based on previous studies, we hypothesized that all of the psychedelics will generously decrease the EEG power and connectivity.

## Methods

### Animals

Experiments were outperformed on adult male Wistar rats (SPF, Velaz s.r.o., Prague, Czechia). The animals weighed 280–300 g at the time of surgery, and 300–350 g at the time of registration. For each experiment, groups of 12 animals were used. The size of the groups was chosen upon the previous experience [41, 42], taking into account the 3Rs principles. Rats were randomly assigned to experimental groups. Rats were kept in pairs in standard plastic breeding containers in an air-conditioned room with a controlled temperature (± 22 °C) and humidity (±40%) with a regular twelve-hour (6:00–18:00) light/dark cycle. Access to standardized food and water was ad libitum. All of the rats were experimentally naive and tested only once. The principles of the National Committee for the Care and Use of Laboratory Animals, CZ, and European Union guidelines (86/609/EU) were adhered to. Ethical approval was given by the National Committee for the Care and Use of Laboratory Animals, CZ.

### Drugs

The following substances were used: psilocin (4 mg/kg dissolved in 2 ml of saline (0.9% NaCl), acidified with 10 μl of glacial acetic acid); LSD (in a form of freebase, 0.2 mg/kg dissolved in 20 μl of 96% ethanol and then adjusted to the required volume of 5 ml by saline (0.9% NaCl w/v), mescaline hydrochloride (100 mg/kg) and 2,5-dimethoxy-4-bromoamphetamine (DOB, 5 mg/kg). The drugs were synthesized and supplied by the Pharmaceutical Faculty of Charles University, Hradec Kralove, Czechia. All of the substances were administered subcutaneously in a dose of 2 ml/kg of animal weight. Saline (0.9% NaCl w/v) was used as a placebo in the control group. The dose levels were established with respect to experimental drugs potencies, as tested using drug-discrimination tests (see Table 1 in ref. [43]). We selected high doses that showed the same effect of locomotion inhibition and deficits in prepulse inhibition (e.g., see our previous studies [19, 44, 45]). The doses used should therefore correspond qualitatively to each other. The investigator was not blinded to the treatment.

### Stereotactic surgery

**Fig. 1:**
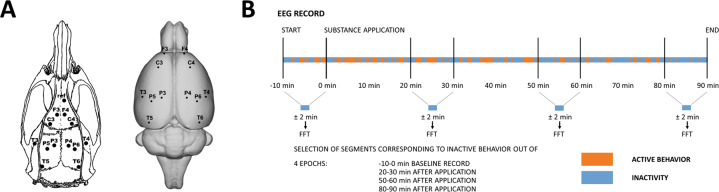
Experimental design. **A** Layout and labeling of the electrodes on the rat’s skull (left) and brain (right). **B** Schematic drawing illustrating the time course of the experiment and selection of signals for further analysis. Four time epochs were selected. Out of each 10-min epoch, only artifact-free EEG signals corresponding to behavioral inactivity segments (in blue) altogether lasting ±2 min were selected and further subjected to Fast Fourier Transform and subsequent analysis.

On the day of surgery, the rats were anesthetized by inhalation of Isoflurane (2.5–3%) and mounted in a stereotactic frame (Stoelting) with atraumatic ear-bars. After shaving the head and disinfecting the operating field with betadine, scissors were used to incise the oval area of skin and the periosteum was cleared off the skull. Coordinates were taken from the Paxinos Rat Brain Atlas [46] (A + 5 mm and L ± 2 mm for the frontal association cortex (electrodes F3/F4), A + 2.2 mm and L ± 3.2 mm for the primary motor cortex (electrodes C3/C4), A − 3.8 mm and L ± 2.5 mm (electrodes P3/P4) for the medial parietal association cortex, A −4.5 mm and L ± 4.5 mm (electrodes P5/P6) for the lateral parietal association cortex, A −3.6 mm and L ± 7.2 mm for the secondary auditory cortex (electrodes T3/T4), and A −8.3 mm and L ± 5.8 mm for the temporal association cortex (electrodes T5/T6)) and calculated according to the established bregma. For details see Fig. 1. The holes were drilled with a stand drill (Stoelting). We used 0.5 mm wide micro drills manufactured by Meisinger. Gold-plated electrodes (Mill-Max, 0.48 mm wide tail) were inserted into the holes so that they did not extend beyond the inner edge of the skull (i.e., epidurally). To the additional number of twelve electrodes of our regions of interest, a reference electrode was implanted above the olfactory bulb (in the same way as the other electrodes) and a ground electrode was subcutaneously inserted under the skin in the occipital region. All of the electrodes were fixed to the skull with Dentalon dental cement. The whole procedure took 40 min on average. Postoperatively, the rats were treated with ketoprofen (5 mg/kg, s.c.). The rats recovered from the anesthesia within a few minutes and often started to eat immediately. All of the rats tolerated the surgery well. Animal body weights were recorded daily. None of the rats tended to lose weight. After the surgery, the rats were individually separated (to prevent biting of the implant) until the day of registration. One day before the experiment, a connector was mounted to the electrodes under short-term anesthesia and fixed with dental cement.

### EEG recording

The experiments were conducted during the daytime, between 7:30 and 13:00 7 days after surgery. Approximately 15 mins before the experiment began, the animals were connected to the EEG system in their home cages. A 10-min recording session was obtained immediately before treatment to serve as a baseline condition. Subsequently, treatments were administered, and registration continued for another 90 min. The rats were able to move freely in the cage during all of the EEG recording sessions while being connected with a cable to the amplifier. Raw EEG signals were recorded using the BrainScope (M&I, Prague, Czechia) data acquisition system with a 16-bit depth, 7.63 nV/bit resolution (i.e., ∼130 bit/μV) and a dynamic range of ±500 μV. The data were recorded with a sampling rate of 250 Hz. EEG data were stored on a PC hard disk for offline processing and analysis. Each rat was recorded only once. In parallel with the EEG data recording, two types of behavioral activity (active behavior/inactivity) were scored (blindly to treatment) by an experienced observer. The animals were handled for a few seconds if a suspicion of sleep was observed (i.e., the animals did not move and tended to close their eyes).

### EEG signal preprocessing and analysis

From the 12 operated animals per treatment group, 10 rats for saline, nine rats for LSD, eight for psilocin, 12 for mescaline, and 11 for DOB were selected for signal analysis, the others were excluded due to technical difficulties during the recording and/or insufficient data quality. We have already shown that psychedelic effects on QEEG in rats are congruent among active/inactive behavior [47]; however, spectral power has been shown to be altered by rat behavior [48]. To prevent of behavioral bias and also to keep our results translational, we used only segments corresponding to behavioral inactivity for further processing. We consider signal analysis from wakeful inactive segments of behavior to be a solid model of resting-state-like EEG recording. Initially, using WaveFinder 2.3. software (M&I, Prague, Czechia), the EEG data were bandpass filtered with a linear FIR (Finite Impulse Response) filter with 111 coefficients in the range of 0.5–45 Hz and then segmented according to behavioral activity and inactivity scoring. To see the dynamics of the changes induced by treatments, we analyzed four distinct time epochs: (1) 0–10 min before treatment—baseline record; (2) 20–30 min post treatment; (3) 50–60 min post treatment; and (4) 80–90 min post treatment. Segments corresponding to behavioral inactivity within each of these time epochs were exported as a quasi-continuous signal for further processing. For details, see the scheme in Fig. 1. Each 10-min EEG segment was then subjected to editing using the Neuroguide software (Neuroguide^©^ NG-2.4.6, Applied Neuroscience Inc., St. Petersburg, FL). Data were preprocessed in the same way as in our previously published studies [41, 42]. Finally, all characteristic artifact-free EEG patterns with minimum lengths of 50 s were used for subsequent analysis. The selected signal was tested for reliability according to Thatcher et al. [49] and then subjected to Fast Fourier Transform.

Absolute power spectra analyses were performed directly by the Neuroguide software. Spectra within the range of 0.5–40 Hz were calculated at a 0.5-Hz resolution for each 2-s epoch. Auto-spectral power was calculated as a diagonal of the cross-spectral matrix (square root of the sums of squares of the real and imaginary coefficients), where the imaginary coefficient is zero and power is sine square. For the coherence analysis, the same signal was exported from Neuroguide to ASCII and then analyzed in Matlab using the Fieldtrip toolbox. Coherence was computed as an absolute value of coherency (cross-spectral coherence): the square of the cross-spectrum divided by the product of the two auto-spectra. Furthermore, we analyzed the imaginary part of coherency (phase-lagged coherence), which is a conceptually accepted solution for tracking the true interactions of the sources underlying the two time series across distributed and distant brain regions, as a given index of correlation cannot be caused by a linear mixing of uncorrelated common sources (volume conduction) [50–54]. Based on the presumed homologous frequency bands in humans [55], analysis was performed on the following frequency bands: delta (1–4 Hz), theta (4–8 Hz), alpha (8–12 Hz), beta (12–25 Hz), high beta (25–30 Hz), and gamma (30–40 Hz). Coherence was derived from 30 intra-hemispheric electrode pairs (F3–C3, F3–P3, F3–P5, F3–T3, F3–T5, C3–P3, C3–P5, C3–T3, C3–T5, P3–P5, P3–T3, P3–T5, P5–T3, P5–T5, T3–T5 on the left hemisphere and analogically on the right) and six inter-hemispheric electrode pairs (between electrodes F3–F4, C3–C4, P3–P4, P5–P6, T3–T4, T5–T6).

### Statistics and visualization

Due to high interindividual variability, the computed data were normalized by the Box-Cox Ratio transformation with *λ* = 0 [56, 57] and then tested by Shapiro–Wilk’s test to ensure normality. Analysis of variance for repeated measures (RM-ANOVA) with the Greenhouse–Geisser correction was used in order to compare the effect of specific treatment on the mean (averaged over all electrodes) spectral power or coherence (between-subject factor) and in time (within-subject factor). Each frequency band was assessed separately. The significance level was set to *P* < 0.05. Post hoc tests corrected for multiple comparisons by the Bonferroni method were then applied in cases of the statistically significant interactions between factors “treatment” and “time”. The Matlab built-in statistical toolbox was used for all of the statistical analyses. Mean values of absolute EEG power spectra difference were plotted on graphs using the GraphPad Prism 8 software. Topographic maps depicting the distribution of significant spectral power change were created using the method of 3D spline mapping [58]. Statistical evaluation was performed separately for each point of the map. Matlab in-house built scripts were used for topographical mapping and visualization. Coherence visualizations were performed in the Python software. The raw effect is depicted by the line width, and the varying colors are the differences between the group means of coherences after the Box-Cox correction. The higher the group mean of the Box-Cox-corrected coherences, the higher the mean relative difference from the baseline in a group. The scale for depicting coherence was set with respect to the maximal and minimal differences across all conditions. We characterized a global connectivity pattern by plotting a probability distribution of all relative connectivity changes from the baseline. Each distribution was obtained by the Kernel Density Estimator (KDE) applied to concatenated coherence values across all subjects, electrode pairs, and frequency bands. The Gaussian kernel of 0.5 width was used in this case. Pie charts depicting the behavioral activity of the rats are a plot of the average ratio (group per time epoch) of time spent actively/inactively as scored during the EEG recording.

## Results

The behavioral activity was assessed as the ratio of active behavior to inactivity in each 10-min epoch. One-way ANOVA showed no difference between the baselines of treatments. Despite the significant interaction revealed by repeated-measures ANOVA between time and treatment (*F*(8,86) = 2.29, *P* < 0.05), Tukey’s post hoc analysis showed no significant effects between the control group and the active substances at the appropriate times.

### EEG absolute mean power spectra

Repeated-measures ANOVA on power spectra revealed a significant effect of treatment in all frequency bands, specifically, for delta (*F*(8,86) = 4.95, *P* < 0.0001), theta (*F*(8,86) = 8.11, *P* < 0.00001), alpha (*F*(8,86) = 5.69, *P* < 0.00001), beta (*F*(8,86) = 4.83, *P* < 0.0001), high beta (*F*(8,86) = 4.76, *P* < 0.0001), and gamma (*F*(8,86) = 7.08, *P* < 0.00001) frequency bands.

**Fig. 2:**
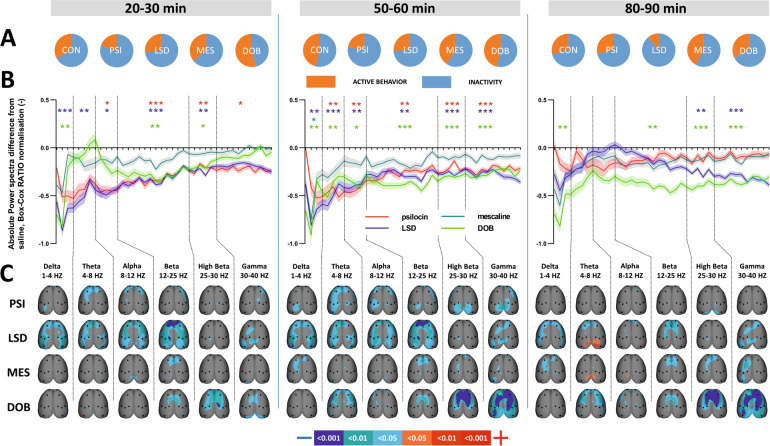
The effects of treatments on EEG power spectra in three time epochs. The abbreviations are given as follows: CON (control group, saline 2 ml/kg), PSI (psilocin, 4 mg/kg), LSD (lysergic acid diethylamide 0.2 mg/kg), MES (mescaline 100 mg/kg), DOB 5 (2,5-dimethoxy-4-bromoamphetamine, 5 mg/kg). **A** Pie charts depicting behavioral activity—average time the rats spent active (orange) and inactive (blue). **B** Mean values of absolute EEG power spectra difference against the control group normalized by the Box-Cox method for all substances over consecutive time epochs; the *x* axis shows the frequency in Hz, the *y* axis the dimensionless quantity. The level of significance is indicated as *P < 0.05, **P < 0.01, ***P < 0.001. Lines show the mean values, shaded error bars correspond to standard error mean values. **C** EEG topographic maps of absolute power spectra differences—all treatments against the control group, drugs in rows, frequency bands in columns; only a significant change is displayed. The direction of change is indicated by a change in color—decreases in blue, increases in red. The level of significance is indicated by a three-level-scaled color spectrum, see the legend. Corresponding legends are at the bottom of each panel.

All of the psychedelics, with the exception of mescaline, resulted in a power decrease within the whole evaluated frequency range of 1–40 Hz. Post hoc analyses revealed the most significant changes (*P* < 0.0–0.001) in the time window 50–60 min after administration, less during the onset of their action at 20–30 min, and only DOB and LSD effects remained after 80–90 min. For details, see Fig. 2.

### Topographic maps of EEG power spectra

Figure 2C shows the 3D EEG statistical topographic maps compared to the controls over the cortical surface for different frequency bands and three consecutive time epochs, treatment by treatment. The maps depict spatial distribution patterns of power spectra differences. All of the drugs induced overall decreases in EEG activity, which were most prominent during the time window of 50–60 min for all treatments, and pronounced for psilocin and LSD after 20–30 min and in the case of mescaline and DOB after 80–90 min. While tryptamine derivatives (psilocin and LSD) induced major decreases on large cortical areas covering the fronto-parieto-temporal cortices and avoided midline structures, phenethylamine derivatives (mescaline and DOB) also induced changes in frontal midline structures. Psilocin and LSD were effective within the lower frequencies (1–25 Hz; delta to beta), whereas mescaline and DOB were more effective at higher frequencies (25–40 Hz; high beta and gamma). Minor increases in theta and alpha power in the temporal and visual cortices for LSD and mescaline appeared in the last epoch.

#### Coherence

**Fig. 3:**
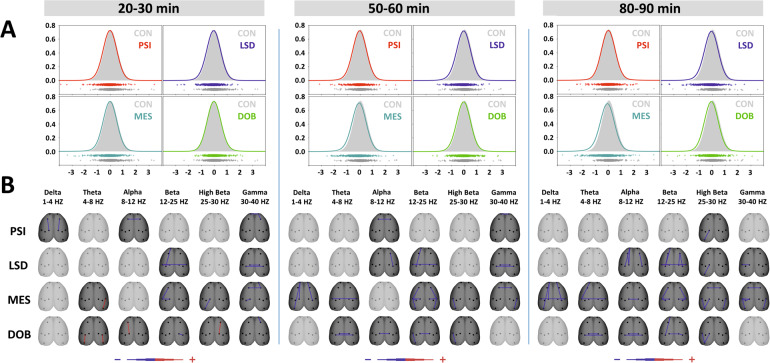
The effects of treatments on cross-spectral coherence in three time epochs. **A** Global cross-spectral coherence connectivity. Each figure contains estimated probability distributions of connectivity in a sense + /− (increase decrease) following drug treatment (solid line) vs control (filled area) condition. The curve is representing the reduced dimensionality of global connectivity generated from all coherence between all electrode pairs across all frequency bands and all animals into 2D space. A negative or positive change of global functional connectivity in a drug condition is indicated by its shifted distribution toward negative or positive values with respect to the control condition. In the scatterplots below each graph, each individual dot represents one value of individual functional connectivity of one subject/electrode pair/in a defined frequency band obtained by the Kernel Density Estimator (KDE). CON (control group, saline 2 ml/kg, gray), PSI (psilocin, 4 mg/kg, red), LSD (lysergic acid diethylamide 0.2 mg/kg, blue), MES (mescaline 100 mg/kg, teal), DOB 5 (2,5-dimethoxy-4-bromoamphetamine, 5 mg/kg, green). **B**, EEG cross-spectral coherence topographic maps—all treatments compared against the control group, drugs in rows, frequency bands in columns; Only significant change is displayed (*P* < 0.05), the direction of change is indicated by the color—decrease in blue, increase in red. The level of relative difference is indicated by a three-level-scaled line width < −3, −2, −1, 1, 2, 3 > , see the legend.

**Fig. 4:**
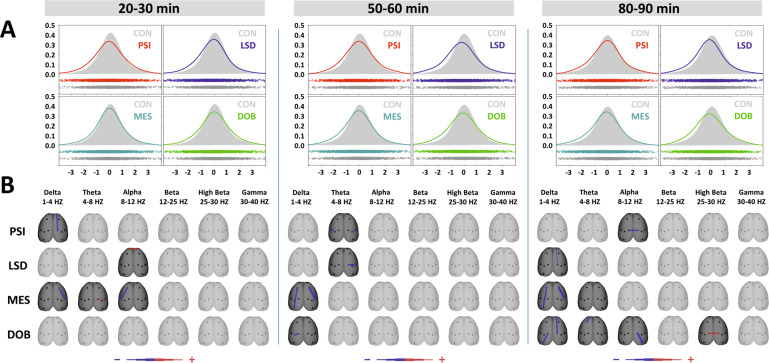
The effects of treatments on phase-lagged coherence in three time epochs. **A** Global phase-lagged coherence connectivity. Each figure contains estimated probability distributions of connectivity in a sense + /− (increase decrease) following drug treatment (solid line) vs control (filled area) condition. The curve is representing the reduced dimensionality of global connectivity generated from all coherence between all electrode pairs across all frequency bands and all animals into 2D space. A negative or positive change of global functional connectivity in a drug condition is indicated by its shifted distribution towards negative or positive values with respect to the control condition. In the scatterplots below each graph, each individual dot represents one value of individual functional connectivity of one subject/electrode pair/in a defined frequency band obtained by the Kernel Density Estimator (KDE). CON (control group, saline 2 ml/kg, gray), PSI (psilocin, 4 mg/kg, red), LSD (lysergic acid diethylamide 0.2 mg/kg, blue), MES (mescaline 100 mg/kg, teal), DOB 5 (2,5-dimethoxy-4-bromoamphetamine, 5 mg/kg, green). **B**, EEG phase-lagged coherence topographic maps—all treatments compared against the control group, drugs in rows, frequency bands in columns; Only significant change is displayed (*P* < 0.05), the direction of change is indicated by the color—decrease in blue, increase in red. The level of relative difference is indicated by a three-level-scaled line width < −3, −2, −1, 1, 2, 3 > , see the legend.

Probability distribution graphs in Figs. 3A and 4A demonstrate the global connectivity patterns by comparing drug and control distributions of all coherence changes. While a control condition resulted in symmetrical distributions around zero, all active substances pull the course of the curve to the left to negative values. Distribution graphs of phase-lagged coherences are generally more scattered and less uniform than those of cross-spectral coherence.

Coherence topographic maps in Figs. 3B and 4B show discrete changes of coherence between electrode pairs over a cortical surface for different frequency bands and three consecutive time epochs. Repeated-measures ANOVA revealed that all treatments induced a decrease in synchronization. While changes in cross-spectral coherence affected the entire frequency spectrum, phase-lagged coherence analysis indicated decreases mainly in the lower frequency bands. Also, phenethylamine derivatives were shown to be more potent in inducing connectivity changes compared to tryptamine derivatives.

## Discussion

Our findings revealed that all psychedelics, irrespective of their structural family, induced a global decrease in the absolute spectral power across the whole of the tested frequency range (1–40 Hz). In addition, at later stages we observed a rebound of theta (4–8 Hz) activity above the occipital visual cortex following treatment with LSD and mescaline but not for the other drugs. All of the substances also considerably decreased brain connectivity.

The magnitude and dynamics of EEG power change were in line with the data on pharmacokinetics published elsewhere [19, 59–61]. Psilocin as a drug with a short biological half-life induced the most pronounced changes during the first epoch and by the last epoch, the effect almost vanished. In contrast, the effects of LSD, mescaline, and DOB persisted until the end of the testing period; DOB had the most pronounced effects in the last testing window and also had a slower onset compared to the other substances.

While the overall image of changes clearly shows that all of the psychedelics induced a decrease in power across the tested frequencies, in the case of LSD and mescaline a biphasic effect with increases in the theta (4–8 Hz) and also partly in alpha (8–12 Hz) band above the visual cortex was observed. As theta and alpha are the dominant resting-state activity in rats [62], we in fact observed a rebound of these activities. Within the pharmaco-EEG studies with related substances, similar increases are typical for stimulants [41, 42, 63, 64]. At the same time, LSD has been shown to have a time-biphasic behavioral effect: the first phase lasting approximately an hour is mediated by 5-HT2A receptors, and the second phase that follows is mediated by D2 and D4 receptors [65–67]. Indeed, D2 and D4 receptor agonist promotes a EEG power increase within the frequency range of the alpha band (8–12 Hz) [68, 69]. In our previous study, we also showed that mescaline had potent stimulatory locomotor effects in a novel environment [19]. Therefore, we may hypothesize that the power increases are linked to the stimulatory effects of these drugs.

Compared to the other substances, DOB during the 20–30 min window had a minimal impact on mean activity within the theta and alpha bands (4–12 Hz) and in contrast showed a tiny peak at 8 Hz. Taking into account that DOB had a high proportion of behavioral activity at this time window, it may correspond to the characteristic peak induced by arousal or to general stimulative properties typical of phenethylamines [4, 70]. Interestingly, an analogous peak of activity contrasting the overall decrease was also seen after treatment with the most closely related compound DOI [48, 71]. Compared to the other substances, DOB-induced decreases in activity were most prominent in the high beta and gamma bands. This is surprising considering the fact that DOB is significantly more selective for 5-HT2A receptors over 5-HT1A [72] compared to other treatments [73] and based on the findings on gamma activity modulation with 5-HT2A and 5-HT1A antagonists rather the opposite effect would be expected [74].

Early animal studies investigating changes in EEG signal under psychedelic intoxication are methodologically disparate and were mostly evaluated by visual inspection [32–36]. However, the most recent animal studies recording local field potentials (LFPs) after treatment with various psychedelics revealed similar effects [37–40]. Human clinical data also consistently show decreases in activity with the most pronounced effects within the alpha band and occipital regions of the visual cortex and related associative areas. Given that psychedelics in humans typically induce perceptual changes, it was proposed that the disruption of the alpha rhythm may be connected with altered visual processing [31, 75, 76]. As visual processing is not the primary source of information about a rat’s reality [77], it is possible to expect broader sensory areas to be affected as seen in our study. In contrast to several human studies [27–29], but in line with others [21–26, 31] as well as preclinical data [39, 40], we also showed a robust decrease within the high beta and gamma ranges. In contrast to typically increased muscle contamination of human EEG following psychedelics [30], all of these animal studies are devoid of muscle artifacts due to recordings being taken directly from the cortex. Therefore, we may clearly assume that it is a real effect on brain activity. Taken together, all of the substances induce broadband desynchronization in a similar way as has been shown in humans for psilocybin and LSD using EEG or MEG [22–25, 31] and are also in line with early EEG human studies (reviewed in ref. [21]), indicating a robust translational validity of the data.

Despite the fact that comparisons with other findings on connectivity in animals are limited, at least referring to a few previous studies, we are able to see a similar pattern, a general decrease in connectivity in animals following treatment with phenethylamine psychedelics 4-bromo-2,5-dimethoxyphenethylamine (2C-B; at doses that induced inhibition of locomotor activity) and DOI, as well as in ketamine-treated animals (in epochs of behavioral inactivity) [39, 41, 42]. Therefore, we propose that after the global desynchronization of EEG activity, the observed general decrease in connectivity is the second epiphenomena of psychedelic effects in animals.

Studies in human volunteers also show alterations in connectivity; however, as they use more advanced methods focusing on large-scale networks these data are hard to compare. Inasmuch it seems that the disruption of stability and integrity of major large-scale brain networks [24, 75] is one of the typical signs having the same direction of changes as may be seen in our data. Future challenges using methods of source localization in rats [78] will bring us to a point where connectivity analyses will be more translational.

Finally, taken together, our study is the first to show a direct comparison of several psychedelics with such consistent results and translational validity. This opens up validity and usability in uncovering the neurobiology of psychedelic effects e.g., using direct modulation of target receptors, or studying mechanisms related to their therapeutic potential, e.g., in animal models of depression.

## Conclusions

We have demonstrated that all psychedelics were associated with specific electrophysiological changes characterized by decreased overall activity and also decreased global connectivity. This is completely in accordance with human EEG findings where similar results were obtained. Furthermore we showed that there are only modest differences between tryptamine and phenethylamine-derived psychedelics. Our data depict the strong translational validity of our model.
